# Characterization of ZnO nanoparticles synthesized using probiotic *Lactiplantibacillus plantarum* GP258

**DOI:** 10.3762/bjnano.16.8

**Published:** 2025-01-30

**Authors:** Prashantkumar Siddappa Chakra, Aishwarya Banakar, Shriram Narayan Puranik, Vishwas Kaveeshwar, C R Ravikumar, Devaraja Gayathri

**Affiliations:** 1 Department of Studies in Microbiology, Davangere University, Davangere, Karnataka, 577007, India; 2 Central Research Laboratory, S.D.M College of Medical Sciences and Hospital, Shri Dharmasthala Manjunatheshwara University, Dharwad, Karnataka, India; 3 Research Centre, Department of Science, East West Institute of Technology, Bangalore, 560091, India

**Keywords:** anticancer, green synthesis, lactic acid bacteria, nanoparticles, zinc oxide

## Abstract

The fundamental goal of our investigation is to employ a sustainable synthesis method for zinc oxide nanoparticles (ZnO NPs), utilizing lactic acid bacteria isolated from curd as the key biological agent. Bacteria function as agents for both reduction and capping processes, which aids the synthesis of ZnO NPs. Various characterization techniques including XRD, FTIR, UV–vis, TEM, SEM-EDX, and zeta potential measurements were employed to analyze the morphology, dimensions, and elemental composition of the generated nanoparticles. The experimental outcomes confirmed the presence of hexagonal wurtzite-structured ZnO NPs with an average size of 10 nm. The colloidal system demonstrated excellent stability with a zeta potential of −60 mV. Furthermore, the synthesized nanoparticles displayed significant antibacterial activity against selected human pathogens, with the biggest inhibition zone observed against *Staphylococcus aureus* (22 ± 0.57 mm) and the smallest inhibition zone observed against *Salmonella enterica serovar typhi* (3 ± 1 mm). MTT assay revealed the promising antiproliferative potential of ZnO NPs, with an average IC_50_ value of 98.53 µg/mL. Additionally, the NPs were photocatalytically and electrochemically analyzed, indicating their potential use in cancer research as well as in coating and drug delivery applications.

## Introduction

Nanotechnology has revolutionized various fields through its remarkable development and the unique properties exhibited by nanoparticles (NPs) at the mesoscopic level. Dimension, form, surface-to-volume ratio, and magnetic, electrical, optical, antimicrobial and hardness properties give NPs distinct mechanical, thermal, and catalytic properties. As a result, nanotechnology has widespread applications across diverse domains and opened up new possibilities for innovation [1–2]. Particles with a size below 100 nm are generally considered NPs. There are several drawbacks to the chemical and physical production of NPs. Although chemically synthesized NPs are widely used in medical applications, some chemical synthesis methods involve toxic reagents and produce harmful byproducts, raising concerns about environmental and biological compatibility. In contrast, green synthesis methods, which use biological agents, such as plants, algae, fungi, and bacteria, under milder, more sustainable conditions offer a more eco-friendly and biocompatible approach to NP production, especially for biomedical applications. This work reports on the green synthesis of ZnO NPs employing lactic acid bacteria (LAB) as capping and reducing agents to produce biocompatible NPs for use in pharmaceutical and biological applications [3–5].

LAB are Gram-positive, catalase-negative, and non-pathogenic bacteria. When ingested in sufficient quantities, they provide health advantages as they colonize the gut, and their metabolites generally show antimicrobial and health promotion activity to the host [6–8]. The thick peptidoglycan layers with crosslinks and the complex cell wall structures of Gram-positive bacteria contribute to the creation of NPs by acting as ligands for the metal ions. Extracellular and intracellular enzymes also play a role in this process, acting as capping, stabilizing, and reducing agents [9].

The study focuses on using LAB to synthesize ZnO NPs with wurtzite (B4) structure under ambient conditions with a bandgap and high exciton binding energy of 3.37 eV and −60 meV, respectively [10]. Because of this high exciton binding energy even at room temperature, the excitonic transitions have a broad range of applications such as in optics, gas detecting, piezoelectrics, and semiconductors. Also, ZnO NPs exhibit antimicrobial activity, targeted drug delivery, catalytic activity, and antidiabetic, larvicidal, acaricidal and anticancer activity in addition to their usage in different medical devices and pharmaceuticals [11–13].

We report the ecologically safe production of ZnO NPs with LAB, which were isolated from curd samples from the rural areas of the North Karnataka region and identified using 16s rRNA sequencing. The efficient synthesis of ZnO NPs indicates the reducing and capping ability of the bacterial isolates. The synthesized NPs further underwent characterization using UV–vis spectroscopy, Fourier-transform infrared spectroscopy, X-ray diffraction measurements, energy-dispersive X-ray spectroscopy, scanning and transmission electron microscopy, photocatalytic studies, electrochemical analysis, and determination of antibacterial and anticancer activity.

The major objective of the present work was to isolate indigenous LAB from rural areas, where fermented foods are produced using traditional methods, the consumers showed better health status and longevity, and the starter culture was maintained for several decades. Our objective was to synthesize ZnO NPs using the indigenous LAB strain and understand its health-promoting characteristics and to extrapolate the beneficial characters to industrial scale [14–15].

## Results

### Physiological and biochemical characterization of LAB

The bacterial isolates from indigenous curd samples were cultured on MRS agar medium and underwent physiological and biochemical characterizations. GP258, a Gram-positive, non-motile, catalase-negative, non-spore-forming, and citrate-negative isolate, was selected for further profiling using carbohydrate fermentation. Eleven different sugars (cellobiose, arabinose, esculin, mannose, melibiose, xylose, maltose, galactose, raffinose, sucrose, and trehalose) were tested. Of all the sugars, trehalose showed variable fermentation. Based on the fermentation profile, it was tentatively identified as *Lactiplantibacillus* and further underwent to molecular characterization.

### Molecular characterization and identification

**Figure 1:**
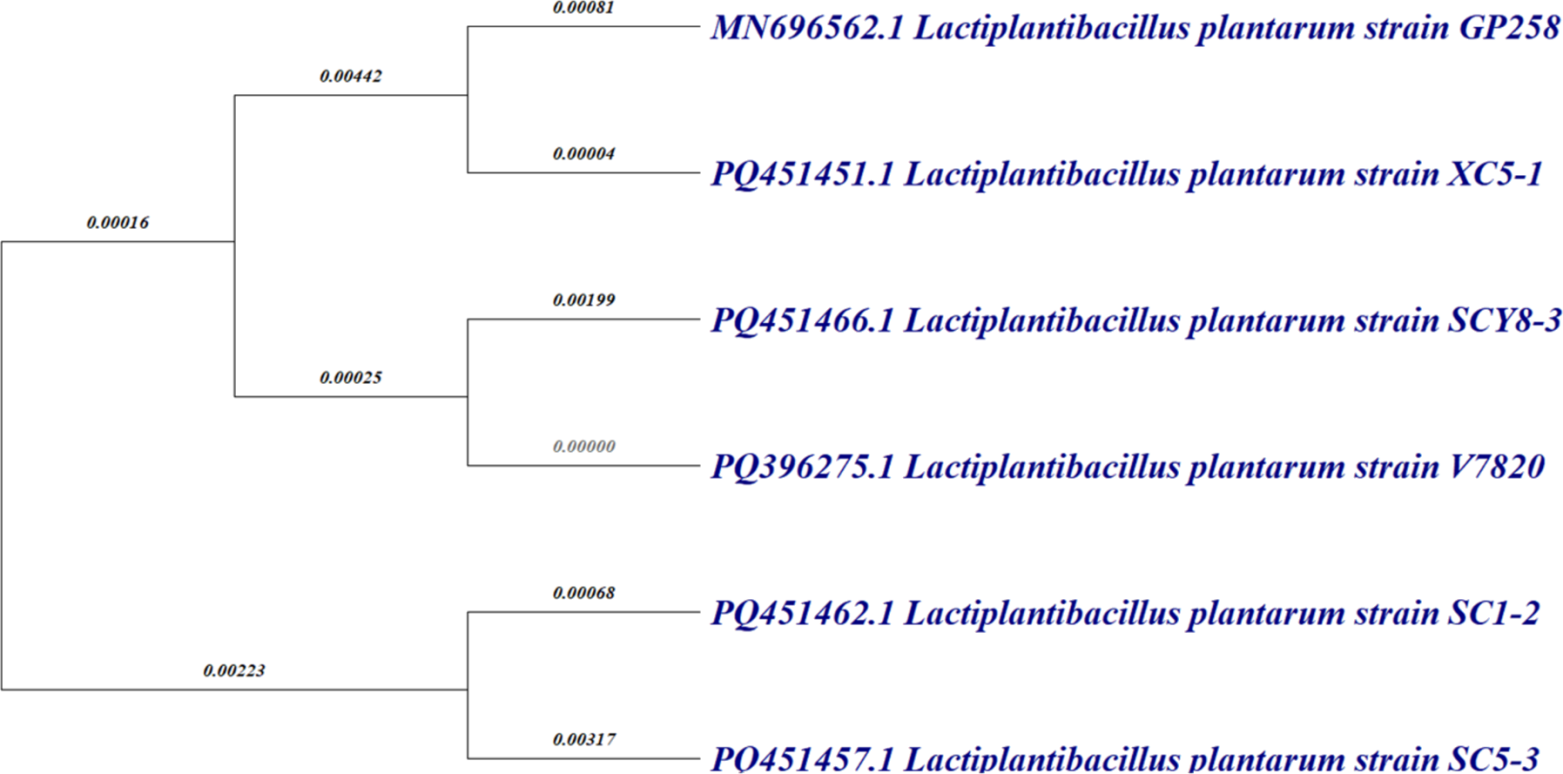
Phylogenetic relationship of GP258 and its evolutionary positioning within the *Lactiplantibacillus* genus. The numbers on the branches represent bootstrap values derived from 1000 replications, showcasing the statistical reliability of each node. The tree was constructed using the maximum likelihood method based on 16S rRNA sequences, with alignment performed against GenBank data.

16S ribosomal RNA (rRNA) gene sequencing and phylogenetic analysis was performed for GP258 and recognized at the level of subspecies. The 16S rRNA genes from the isolated strain are about 1300 and 1500 base pairs long. They were amplified through PCR, followed by sequencing. Subsequently, the resulting nucleotide sequence was submitted and archived in the GenBank database; it was allocated with the accession number MN696562. An online Nucleotide BLAST analysis was conducted within the NCBI platform, resulting in the identification of a comparable sequence. These sequences were then aligned, forming the basis for the reconstruction of a phylogenetic tree. This reconstruction was carried out using the maximum likelihood method and involved employing a bootstrap value of 1000 replications to establish evolutionary relationships among the gathered *Lactiplantibacillus* sequences sourced from the GenBank database. The software utilized for this purpose was MEGA X. Notably, the constructed phylogenetic tree showcased a remarkable 100% affinity with the *Lactiplantibacillus plantarum* phylogenetic tree is presented in Figure 1.

### Synthesis of ZnO from GP258

GP 258 was employed in the biosynthesis of ZnO NPs. The formation of a white precipitate at the flask’s bottom served as an indicator of ZnO NP production.

### XRD analysis

**Figure 2:**
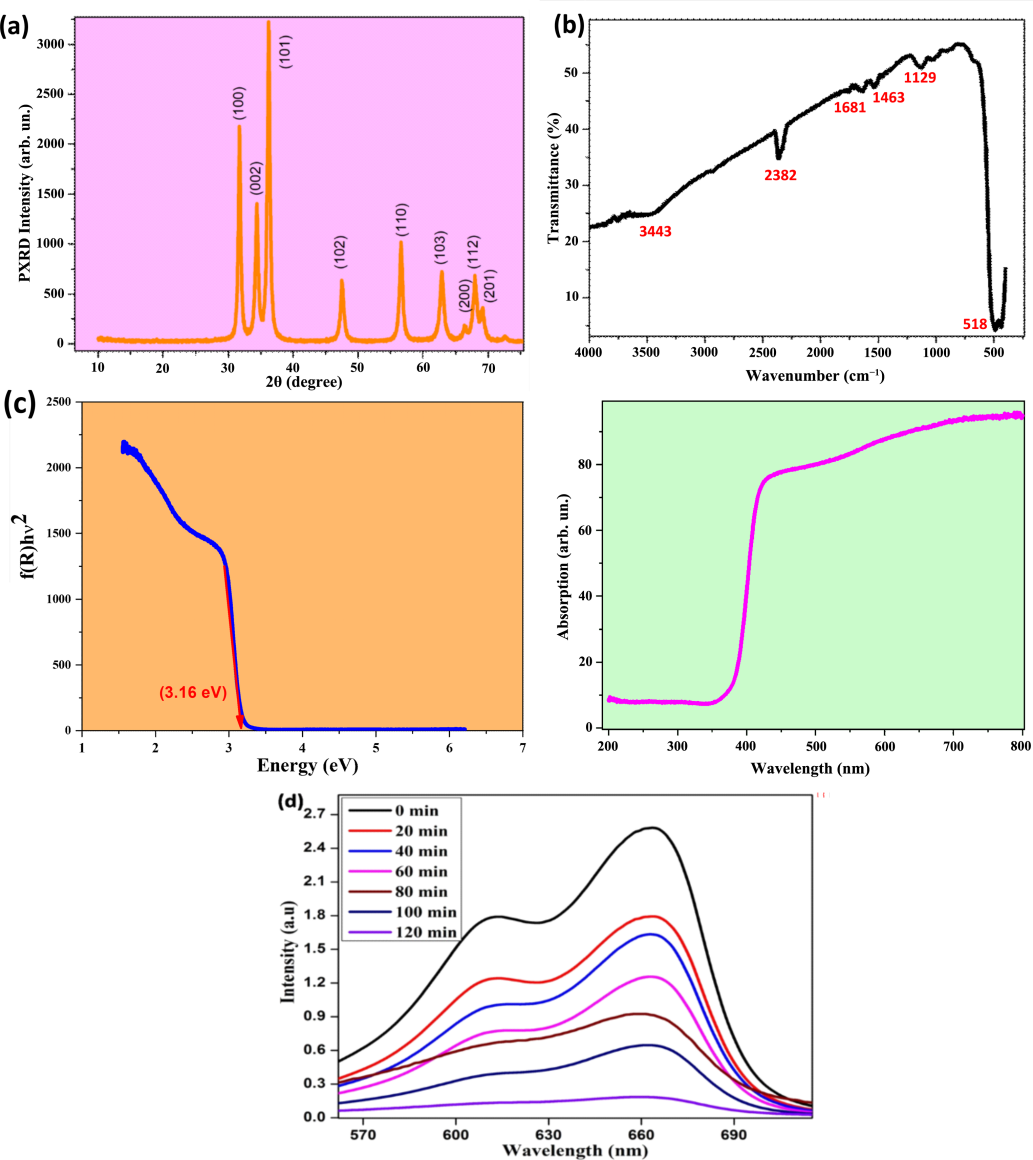
(a) X-ray diffraction analysis, (b) Fourier-transform infrared (FTIR) spectroscopy, (c) UV–vis spectroscopy, and (d) photocatalytic efficiency of ZnO NPs.

The XRD patterns revealed a strong agreement with the hexagonal wurtzite structure, which is characteristic of ZnO NPs. This assertion was substantiated by comparison with data from the JCPDS card no.89-7102. Remarkably, there were no indications of any other phases, indicating a high purity of the ZnO NPs. The XRD reflections were remarkably well-defined and narrow. This signifies the distinctive crystalline arrangement, indicating a robust crystalline structure. The average crystallite size of the synthesized ZnO NPs was calculated using the Scherrer equation:


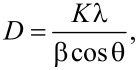


where *D* is the crystallite size, *K* is the shape factor, λ is the X-ray wavelength (1.54 Å for Cu Kα radiation), β is the full width at half maximum (FWHM) of the diffraction peak in radians, and θ is the scattering angle. Using this formula, we determined an average crystallite size of around 25 nm for the 2θ angle of 35.8°. This measurement provided valuable insight into the dimensions of the ZnO NPs and is depicted in Figure 2a.

### FTIR analysis

The FTIR spectra of ZnO NPs were examined in the range from 400 to 4000 cm^−1^. We observed a peak at 484 cm^−1^, which corresponds to Zn–O bond vibrations. In addition, we observed peaks at 440, 480, and 670 cm^−1^ also related to ZnO. The presence of water within the nanocrystalline ZnO NPs was suggested by peaks observed at 3400 cm^−1^, representing O–H stretching vibrations, and at 1626 cm^−1^, indicating H–O–H bending vibrations. Additionally, we found a peak at 2924 cm^−1^, connected to C–H bonds, and peaks between 1400 and 1649 cm^−1^, associated with C=O stretching vibrations. A band around 2335 cm^−1^ could be attributed to CO_2_ absorption on metallic Zn^2+^ cations. These findings offer information about the structure and chemical interactions within the ZnO NPs (Figure 2b).

### UV–vis absorption

The UV–Vis absorption spectra of ZnO NPs, presented in Figure 2c, establish a distinct absorption peak at 3.16 eV, revealing the characteristic bandgap energy for ZnO [16]. This peak, observed in the blue line of the spectrum, confirms the formation of ZnO NPs with semiconductor properties and highlights their high purity, which is proven by the lack of peaks that would indicate impurities. This data validates the successful synthesis of ZnO NPs with a clear electronic transition, making them suitable for further applications in photocatalysis, electronics, and optoelectronics.

### Photocatalytic study

ZnO NPs were utilized in a photocatalytic degradation test to reduce the concentration of the harmful dye methylene blue (MB). A solution containing 20 mg of ZnO NP photocatalyst and 20 ppm of MB dye was exposed to UV light in order to initiate photodegradation. Measurements were taken at 15 min intervals to track the reduction in MB dye concentration. UV–visible spectrophotometry was used to measure the absorbance at 663 nm. Under UV irradiation, the photodegradation of MB dye progressed with time and yielded 95% degradation under 120 min. The process involved in dye degradation is exciting electrons and generating holes in the semiconductor. The produced electrons form superoxide radicals (^•^O_2_^−^) by reacting with O_2_, while holes react with water (H_2_O) molecules to produce hydroxyl radicals (^•^OH^−^). These radicals degrade the dye molecules into harmless substances; the degradation is shown in Figure 2d.

### TEM analysis

**Figure 3:**
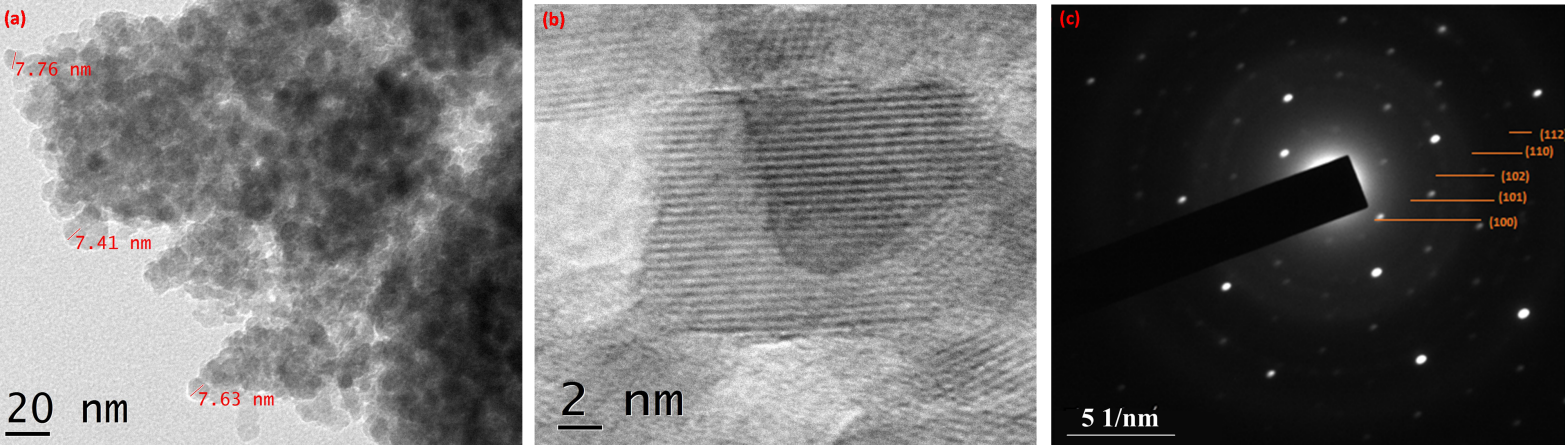
(a, b) Transmission electron microscopy (TEM) micrographs showing the morphology and size of the nanoparticles. (c) Selected area electron diffraction (SAED) pattern of ZnO NPs with orange values representing the Miller indices of the diffraction planes, confirming the hexagonal wurtzite structure.

The size of the ZnO NPs, which was determined from TEM using Image J software, varied from 7 to 98 nm, with an average size of 10 nm. The SAED pattern of ZnO NPs confirms the XRD results, that is, the hexagonal wurtzite structure (Figure 3).

### SEM and EDX analysis

**Figure 4:**
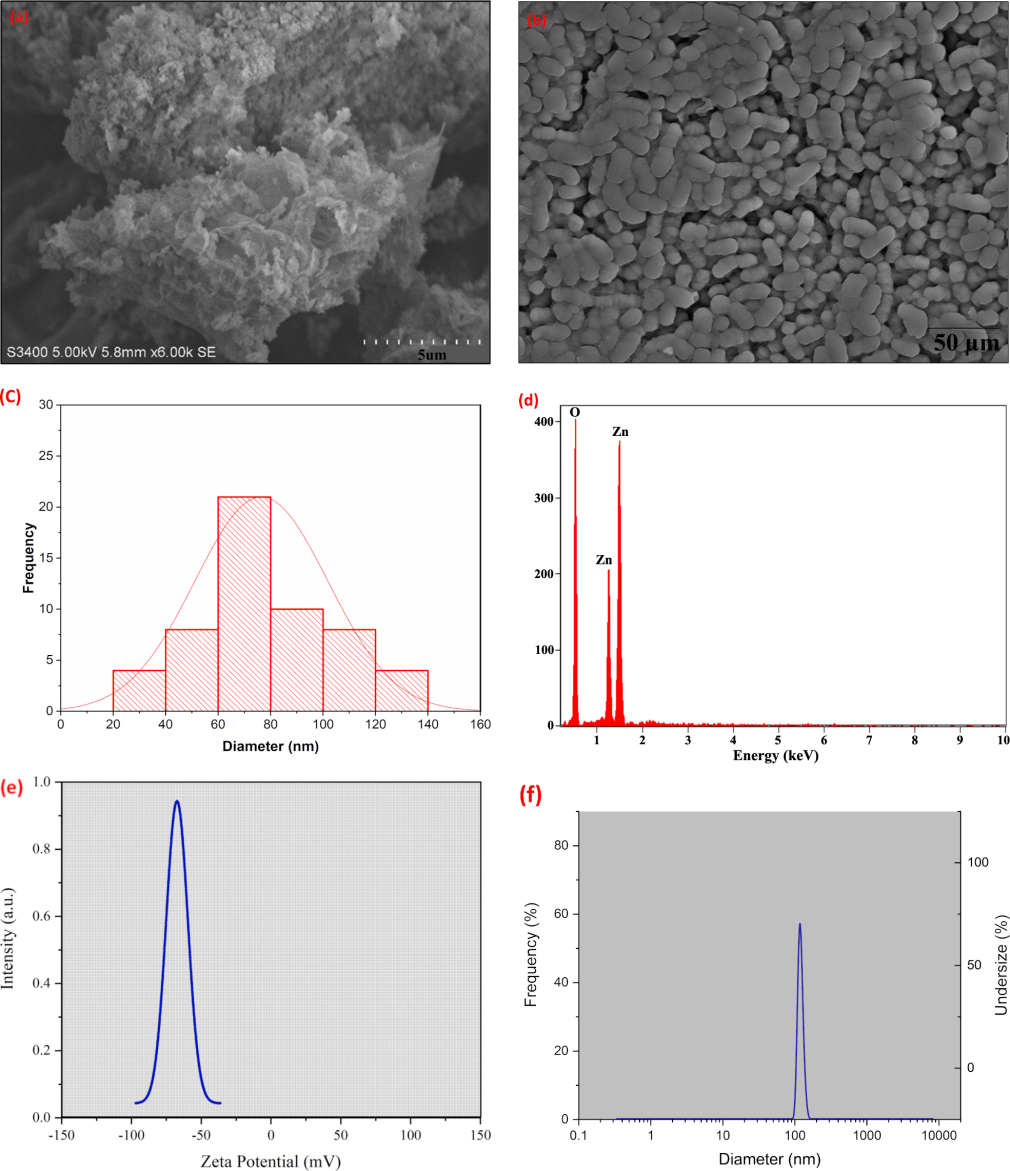
(a, b) Scanning electron microscopy images depicting morphology, structure, and size of ZnO NPs. (c) Frequency distribution of nanoparticle sizes derived from SEM analysis. (d) EDX spectroscopy for elemental composition. (e) Zeta potential measurement. (f) DLS results showing the size distribution of ZnO NPs.

SEM was used to analyze the surface morphology of a modified nanocomposite film, and the image displays a consistent coverage of a web-like structure. Close-up views revealed a crumpled and wrinkled pattern, and it was found that the NPs average size was 72 nm. The presence of ZnO NPs on the surface was confirmed through EDX, which showed characteristic elemental peaks validating the composition (Figure 4a–d).

### Zeta potential

The ZnO NPs synthesized using GP258 showed good stability as colloidal system. Our biosynthesized nanoparticles showed a zeta potential of −60 mV. It is considered that a NP colloid with a zeta potential of more than ±30 mV shows good stability against aggregation. The zeta average size was found to be 99 nm (Figure 4e,f).

### Electrochemical analysis

Cyclic voltammetry (CV) and electrochemical impedance spectroscopy (EIS) were carried out at room temperature using a three-electrode cell with 0.1 M KCl electrolyte. The ZnO NP electrode was measured at scan rates from 10 to 50 mV/s. The measurements revealed reversibility and electrode load efficiency along with reduction currents and increased peak oxidation, indicating rapid electron transport at the contacts between the electrolyte and electrode. Pseudo-capacitive behavior was observed in both electrolytes, where ionic conductivity influenced capacitance [17–18].

**Figure 5:**
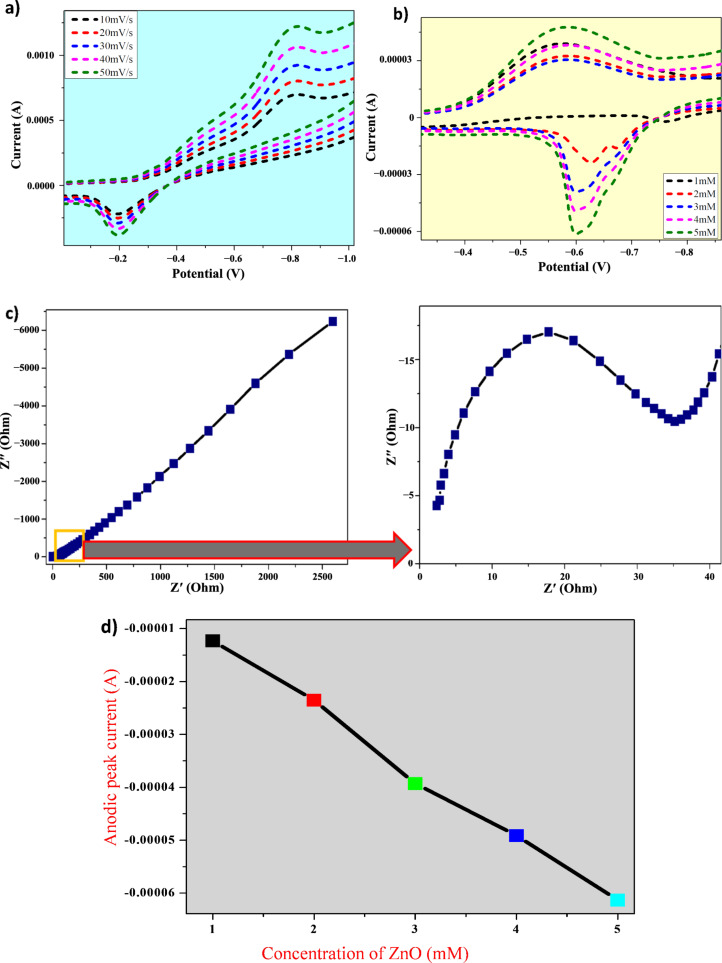
(a, b) Cyclic voltammetry response of the ZnO electrode in 0.1 M KCl solution at varying scan rates, showing redox behavior and electron transfer characteristics. (c) Nyquist plots representing impedance spectra from electrochemical impedance spectroscopy. (d) Concentration vs peak current graph derived from CV studies.

The addition of dextrose increased the redox peak current, though a steady addition led to decreased currents, possibly due to surface accumulation. The Nyquist plots display a larger impedance for ZnO electrodes. To fit the data into an analogous circuit, an uninterrupted phase element (Q1) was introduced with the adjustable parameters angular frequency (ω), *Y*, and *n*. The obtained results suggest robust electron transfer and enhanced electrocatalytic efficiency in dextrose oxidation [19–21] (Figure 5).

### Antibacterial activity of ZnO NPs

**Figure 6:**
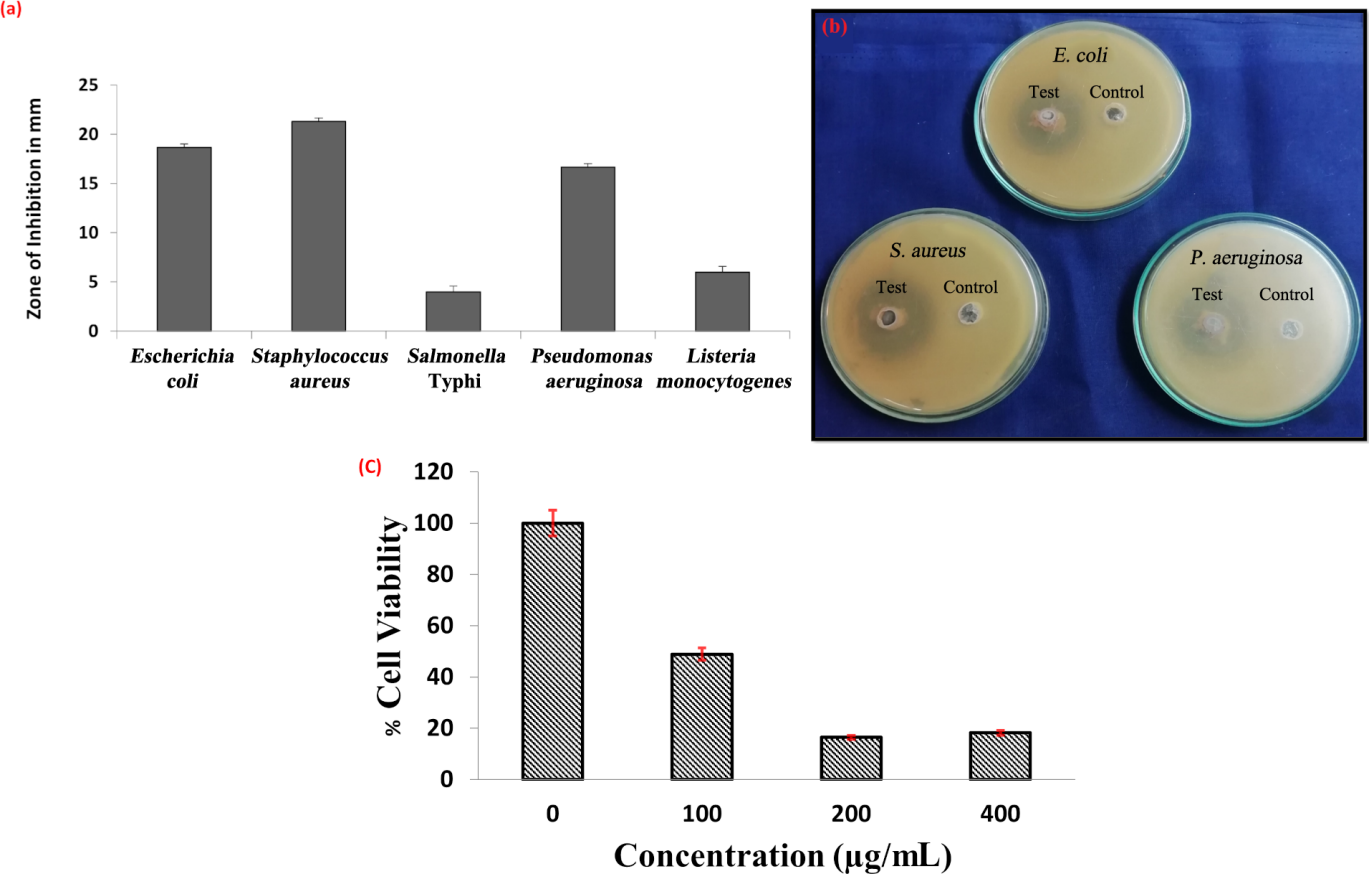
(a) Antibacterial activity assessment against human pathogens showing the zone of inhibition in millimeters. (b) Visual representation of an antimicrobial plate, highlighting the clear zones of inhibition. (c) MTT assay results demonstrating the impact of green synthesized ZnO NPs on HT-29 cells. The data represent the mean ± standard deviation (SD) from three independent experiments, each performed in triplicate.

The biogenic ZnO NPs presented a good dispersion and exhibited antibacterial activity against both Gram-positive and Gram-negative pathogens. Of the five pathogens examined, three exhibited susceptibility to the ZnO NPs, while the remaining two showed reduced zones of inhibition, indicative of resistance. Noteworthy, susceptibility of *Staphylococcus aureus*, evidenced by the largest zone of inhibition measuring 22 ± 1 mm. Subsequently, *Escherichia coli* and *Pseudomonas aeruginosa* exhibited substantial susceptibility, yielding zone diameters of 19 ± 1 mm and 17 ± 1 mm, respectively. In contrast, *Listeria monocytogenes* and *Salmonella enterica serovar typhi* demonstrated resistance, underscored by reduced zone diameters of 6 ± 1 mm and 3 ± 1 mm, respectively. We summarized all these findings visually in Figure 6a,b.

### MTT assay

The anticancer activity of biosynthesized ZnO NPs was assessed, where upon analysis the IC_50_ values of biosynthesized ZnO NPs were evaluated over a period of 24 h at varying concentrations. The IC_50_ values represent the concentration of biosynthesized ZnO NPs required to inhibit the biological response by 50%. The IC_50_ value after 24 h was calculated as 98.53 µg/mL (Figure 6c).

## Discussion

The principal aim of this investigation was to employ an eco-friendly synthesis approach in producing ZnO NPs using indigenous LAB isolated from native breed cow curd from very remote regions of North Karnataka (India), which displayed notable antibacterial activity. Here, people consume curd regularly in their diet. The curd is prepared by traditional methods, and, for several decades, the same starter culture was maintained for curd preparation. People from this region showed better health and longevity and minimum gastrointestinal ailments. To exploit the beneficial health-promoting properties of the probiotic bacteria in these curd samples, the present study was undertaken. The LAB functioned as capping and reducing agents for ZnO NP synthesis, offering a sustainable and environmentally friendly route for nanoparticle fabrication.

To characterize the synthesized ZnO NPs, a range of analytical techniques were harnessed. XRD analysis established the occurrence of ZnO NPs with the hexagonal wurtzite structure. Similarly, in another study, Suba et al. [22] also described the biosynthesis of hexagonal ZnO NPs using *Lactiplantibacillus* spp. Valuable insights into the synthesis process were obtained through FTIR spectroscopy. The peak at 484 cm^−1^ confirms the occurrence of Zn–O linkages. Microscopic examination via TEM and SEM offered details about nanoparticle size and shape. Our study reported a size range from 7 to 98 nm, with an average size of 10 nm. Suba et al. [22] reported an average size of 32 nm, while Mohd Yusof et al. [23] synthesized ZnO NPs with a size of 291 nm (flower-like pattern) and 192 nm (irregular shape).

Valuable insights regarding photocatalytic and colloidal stability of the biosynthesized ZnO NPs was provided by using UV–vis spectroscopy and zeta potential analysis. Compared to a similar type of study conducted by Selvarajan and Mohanasrinivasan [24], our study showed significant photocatalytic activity by degrading methylene blue under UV illumination, achieving a remarkable 74.15% decolorization within 60 min, with a slightly lower energy of UV light, that is, 392 nm whereas above authors reported 385 nm. Our ZnO NPs shows a zeta potential value of −60 mV, indicating robust colloidal stability, contrasting with the moderately negative zeta potential (−15.3 mV) reported by Selvarajan and Mohanasrinivasan [24], which shows possible variations in ZnO NP characteristics, size distribution, surface modification, and experimental conditions. Nonetheless, both studies underscored the efficacy of ZnO NPs in photocatalytic degradation and emphasized their potential applications in environmental remediation and catalysis.

Because there is a lack of cyclic voltammetry studies using similar biological sources, we expanded our comparison to include ZnO NPs synthesized from different biological sources. In our study, cyclic voltammetry was used to assess the electrochemical properties of ZnO NPs, which exhibited reversible redox behavior and efficient electron transfer. A similar study by Matinise et al. [25] on ZnO NPs synthesized using *Moringa oleifera* also demonstrated enhanced electrochemical performance and capacitance modulation. These findings highlight the potential of ZnO NPs as efficient electrocatalysts for various applications in energy-related technologies.

The antibacterial activity of ZnO NPs synthesized using GP258 isolate is likely multifactorial. ZnO NPs are known to generate reactive oxygen species (ROS), including hydroxyl radicals and superoxide ions, upon interaction with bacterial cells. These ROS disrupt bacterial cell membranes, cause oxidative stress, and damage cellular components, ultimately leading to cell death. Additionally, ZnO NPs can release Zn^2+^ ions, which interact with bacterial enzymes and proteins, further compromising cellular functions. The small size and high surface area of the nanoparticles enhance their interaction with bacterial cells, improving antibacterial efficacy. Significant inhibitory effects against *S. aureus* were observed, with a zone of inhibition measuring 22 ± 1 mm. In contrast, *Salmonella enterica serovar typhi* showed lower susceptibility, with an inhibition zone of 3 ± 1 mm, though antibacterial activity was still evident. Suba et al. [22] also reported antibacterial activity of ZnO NPs, demonstrating inhibition against *E. coli*, *C. perfringens*, *C. difficile*, and *Salmonella enterica serovar typhi*. Similarly, Mohd Yusof et al. [23] observed antibacterial effects of ZnO NPs on pathogenic bacteria, underscoring their potential for combating bacterial infections. It is noteworthy that *S. aureus* has the tendency to develop drug resistance, such as MRSA or VRSA [26]. In this context, nanoparticles are perhaps an alternative strategy to control the spread of drug-resistant bacteria. Additionally, research conducted by El-Khawaga et al. [27] showed that ZnO NPs produced by *Saccharomyces cerevisiae* revealed strong antibacterial activity against both Gram-positive and Gram-negative bacteria. Inhibitory zones around the nanoparticles for *S. aureus* were 23.1 mm, and for *E. coli* 17.0 mm. These results indicate that this biogenic synthesis route may yield particles with antibacterial activity against all the given bacteria.

Regarding antiproliferative effects, our study exhibited an average IC_50_ value of 98.53 µg/mL against HT-29 cell lines. In contrast, Mohd Yusof et al. [9,23] evaluated cell viability using the MTT assay on Vero cells, revealing viability at concentrations from 100 µg/mL at 24 h. Noteworthy antiproliferative effects were observed against HT-29 cells at lesser concentrations (54.16%), with an IC_50_ value of 15.6 μg/μL [22], substantiating the potential of the NPs in cancer research and therapy. The synthesized ZnO NPs also underwent photocatalytic and electrochemical analyses, suggesting their potential utility in environmental contexts and as biomedical coatings.

## Conclusion

This work effectively proved the use of native lactic acid bacteria as capping and reducing agents in the green synthesis of ZnO NPs. The NPs exhibited potential structural, optical, and biological qualities that make them usable for a broad variety of applications such as cancer treatments, antimicrobial agents, and cutting-edge materials in several sectors. The sustainable and cost-effective synthesis route, along with the tunable properties of ZnO NPs, offers exciting possibilities for future research and practical implementations. Further investigation is needed to study the exact mechanism responsible for showing antibacterial and antiproliferative effects, paving the way for potential clinical and industrial applications of these biosynthesized ZnO NPs.

## Material and Methods

### Materials

Lactobacillus Man Rogosa Sharp (MRS) Agar, Granulated GM641I, Mueller Hinton Agar, M173, HiLacto™ Identification Kit, KB020, zinc sulfate heptahydrate, Hi-AR™, GRM695, Sure Fetal Bovine Sera, RM10974, Dulbecco’s Modified Eagle Medium (DMEM), AT007, Tissue Culture Flask, TCG6, Tissue Culture Plates 96 wells, TPG96 were procured from Himedia, India and were of analytical grade. The National Centre for Cell Sciences in Pune supplied the human colorectal cancer cell lines (HT-29 ATCC). The Microbial Type Culture Collection and Gene Bank (MTCC, India) provided the test organisms.

### Phenotypic diagnosis

Curd samples were obtained from various locations within the Kalaburagi district, Karnataka, India (17.4047° N, 76.6413° E). These samples were transferred to sterile containers and underwent to serial tenfold dilution with the pour plate technique using MRS agar, prior to incubation at 37 °C for 48 to 72 h. Later, bacterial colonies were screened, and pure cultures were maintained at 4 °C. A comprehensive analytical scheme was adopted to identify the most promising bacterial isolates, that is, Gram’s reaction, morphology, catalase activity, endospore staining, and carbohydrate fermentation profiling using KB020 HiLacto Identification Kit (Himedia, India), following the manufacturer’s instructions.

The selected bacterial isolates were inoculated to 5 mL of MRS broth, incubated (6–18 h at 37 °C) to obtain 0.1 OD (620 nm). Later, 50 µL of the inoculum was aseptically added to each well using the surface inoculation method and incubated for 24 to 48 h at 37 °C. During the incubation period, alterations in color were closely monitored [28–29].

### Identification of selected isolates by 16S rDNA sequencing

The most promising and potential *Lactiplantibacillus* isolate, GP258, was identified by sequencing DNA of 16S rRNA. The CTAB method was used to isolate genomic DNA as per the protocol [30]. Polymerase chain reaction (PCR) was conducted using the following primers: Reverse Primer (396) - 5'-CGGTGTACAAGGCCCGG-3' and Forward Primer (395) - 5'-GGATGAGCCCGCGGCCTA-3'. For the PCR experiments, a thermal cycling procedure was used. Denaturation at 96 °C for 5 min was preceded by denaturation at the same temperature for 30 s, hybridization at 50 °C for 30 s for thirty cycles, with a final elongation step at 60 °C for 90 s. Later, the amplicons were sequenced using the ABI 3130 Genetic Analyzer sequence detection system, following the standard protocol provided by the supplier. The obtained sequences were searched in GeneBank database, and the construction of phylogenetic map was accomplished utilizing MEGA X software [31].

### Production of ZnO nanoparticles

A pure culture of GP258 was used for synthesizing ZnO NPs according to [24] with slight modifications. The GP258 isolate was inoculated to MRS broth and incubated for 24 h at 37 °C. Further, the mixture was diluted fourfold using sterile distilled water and again incubated for 24 h. The culture broth was adjusted to pH 6 using 1 M NaOH, then to the culture broth 0.1 M ZnSO_4_·7H_2_O was added, and the flask was heated to 80 °C for 10 min. The continuous process of transformation could be confirmed by the development of white precipitate at the bottom of the flask. After that, the flask was incubated for a further 12 h at 37 °C to ensure complete particle deposition. The particle sediment was then filtered, cleaned twice with deionized water, and dried for 4 h at 40 °C.

### Characterization of ZnO nanoparticles

XRD patterns were recorded using a Bruker D8 Advance diffractometer with Cu Kα radiation (λ = 1.54 Å), operating at 40 kV and 30 mA. The scan range was 10°–80° (2θ), with a step size of 0.02° and a dwell time of 1 s per step. Reflectance was studied using UV–vis spectroscopy performed on a Shimadzu UV-2600 double-beam spectrophotometer. Nanoparticles were dispersed in deionized water at a concentration of 1 mg/mL, and the spectra were recorded in the range of 200–800 nm with a resolution of 1 nm. Additionally, chemical characteristics were investigated using FTIR analysis on a PerkinElmer Spectrum 65 spectrophotometer in the range of 400–4000 cm^−1^. ZnO NPs were mixed with potassium bromide (KBr) at a ratio of 1:10 (w/w) to prepare pellets for analysis.

TEM was employed to study the shape, size, and distribution of the ZnO NPs. FESEM analysis showed a web-like structure with defined diameters; EDX spectroscopy confirmed the presence of ZnO NPs on the film surface.

Zeta potential measurements and DLS analysis were conducted using the SZ-100 Horiba Scientific instrument from Japan. ZnO NP dispersions were prepared at a concentration of 1 mg/mL in deionized water and analyzed at 25 °C. Each sample was analyzed in triplicate, with the Zeta potential results expressed as the mean ± standard deviation (SD) and the zeta average calculated to quantify the surface electric charge of the ZnO NPs, assessing their stability. DLS measurements followed with an acquisition time of 120 s per run, providing data for the intensity-weighted size distribution.

The photocatalytic activity was analyzed by studying the degradation of methylene blue in a 20 ppm dye solution containing 60 mg of ZnO NPs. The solution was irradiated with UV light at 392 nm, and aliquots were collected in 15 min intervals for spectrophotometric analysis using the Shimadzu UV-2600 double-beam spectrophotometer.

CV and EIS were conducted using a CHI608E analyzer with a three-electrode setup (carbon paste with ZnO NPs as the working electrode, platinum as the counter electrode, and Ag/AgCl as the reference electrode) in 0.1 M KCl. EIS measurements were carried out in a frequency range of 1 Hz to 1 MHz with a 5 mV AC amplitude, while CV studies were carried out on the same instrument to evaluate the electrochemical properties [13,32].

### Biological activity of nanoparticles

#### Antibacterial activity of ZnO NPs

Agar well diffusion was used to measure the antibacterial activity of the biosynthesized ZnO NPs. The test isolates were belonged to both classes of Gram variants, including *Escherichia coli* (MTCC4296), *Staphylococcus aureus* (MTCC3160), *Salmonella enterica serovar typhi* (MTCC8767), *Listeria monocytogenes*, and *Pseudomonas aeruginosa* (MTCC424). ZnO NPs were suspended in sterile deionized water, and the inoculum of each test bacterial pathogen was cultured overnight in nutrient broth at 37 °C. Selected test bacterial isolates were swabbed onto Nutrient agar with all aseptic precautions and allowed to settle for 2 min. Subsequently, a sterile borer was used to dig agar wells, and 100 µg/mL of biosynthesized ZnO NPs were added to each well. The diameter of the inhibitory zone was measured after the plates were incubated for 24 h at 37 °C. This experimental procedure was conducted in triplicates [23].

#### Cell cytotoxicity assay

A colon cancer cell line (HT-29), obtained from NCCS (Pune, India), was treated with ZnO NPs to check their cytotoxicity by using 3-(4,5-dimethylthiazol-2-yl)-2,5-diphenyltetrazolium bromide (MTT, Sigma-Aldrich, USA). The experiment involved seeding cells at a concentration of 25 × 10^2^ cells per well in 96-well microplates for 24 h. Subsequently, the cells were treated with varying masses of ZnO NPs (ranging from 1 to 400 µg) for 24 h. Following this, 20 μL of freshly prepared MTT solution was introduced to each well, and the resulting absorbance was quantified at 570 nm. This experimental procedure was conducted in triplicates [22,33].

## Data Availability

The data used to support the findings of this study are included within the article and if any additional information is required this will be provided on request.
